# Colon Metastasis From Primary Lung Cancer: A Report of a Rare Case and Review of Diagnostic and Therapeutic Challenges

**DOI:** 10.7759/cureus.98650

**Published:** 2025-12-07

**Authors:** Pedro Averbach, Caroline Cirenza, Pedro Popoutchi, Marcelo Averbach

**Affiliations:** 1 Department of Gastroenterology, Hospital Sírio-Libanês, Sao Paulo, BRA

**Keywords:** colon metastasis, endoscopic mucosectomy diagnosis, gastrointestinal bleeding, gastrointestinal metastases, primary lung cancer

## Abstract

This case report aims to describe the rare occurrence of colon metastasis that originated from primary lung adenocarcinoma, highlighting the diagnostic challenges, therapeutic interventions, and clinical implications. A 62-year-old woman diagnosed with a biphenotypic lung carcinoma (squamous and adenocarcinomatous components) developed gastrointestinal (GI) symptoms. Imaging and endoscopic evaluation revealed a 3 cm sessile polypoid lesion in the ascending colon. Histopathological analysis and immunohistochemical profiling confirmed the lesion as a metastatic adenocarcinoma from the primary lung cancer. The patient underwent successful endoscopic mucosectomy with clear surgical margins, followed by targeted therapy with osimertinib due to the presence of an epidermal growth factor receptor (EGFR) mutation. Thus, this case underscores the necessity of considering metastatic lung cancer in the differential diagnosis of colonic lesions, particularly in patients with a known history of lung cancer. Comprehensive histopathological and immunohistochemical analyses are crucial for accurate diagnosis. The management of such rare metastases requires a multidisciplinary approach, integrating surgical and targeted therapies to improve patient outcomes. This report adds valuable insights into the clinical presentation, diagnostic processes, and therapeutic strategies for atypical metastatic patterns in lung cancer.

## Introduction

Lung cancer, a leading cause of cancer-related mortality globally, is known for its aggressive nature and potential to metastasize to various organs [1]. However, gastrointestinal (GI) tract metastasis is uncommon, with an incidence of 0.2%-1.7% [2-5]. Among the GI tract, the colon is even rarer, often leading to diagnostic challenges and therapeutic dilemmas [6,7]. This case report presents a unique instance of gastrointestinal bleeding caused by colon metastasis originating from primary lung cancer, contributing to the limited but growing literature on this atypical presentation.

This detailed examination of the patient's clinical journey, including diagnostic challenges and treatment outcomes, aims to shed light on this rare clinical entity. The case contributes to the existing literature on lung cancer metastases and underscores the importance of considering atypical metastatic sites in oncological diagnoses and management.

## Case presentation

A 62-year-old woman from São Paulo, Brazil, presented to the emergency department with acute hemoptysis in January 2021. She had a history of chronic disseminated intravascular coagulation and chronic autoimmune hepatopathy managed with prednisone. She also reported significant tobacco use (40 pack-years).

**Figure 1:**
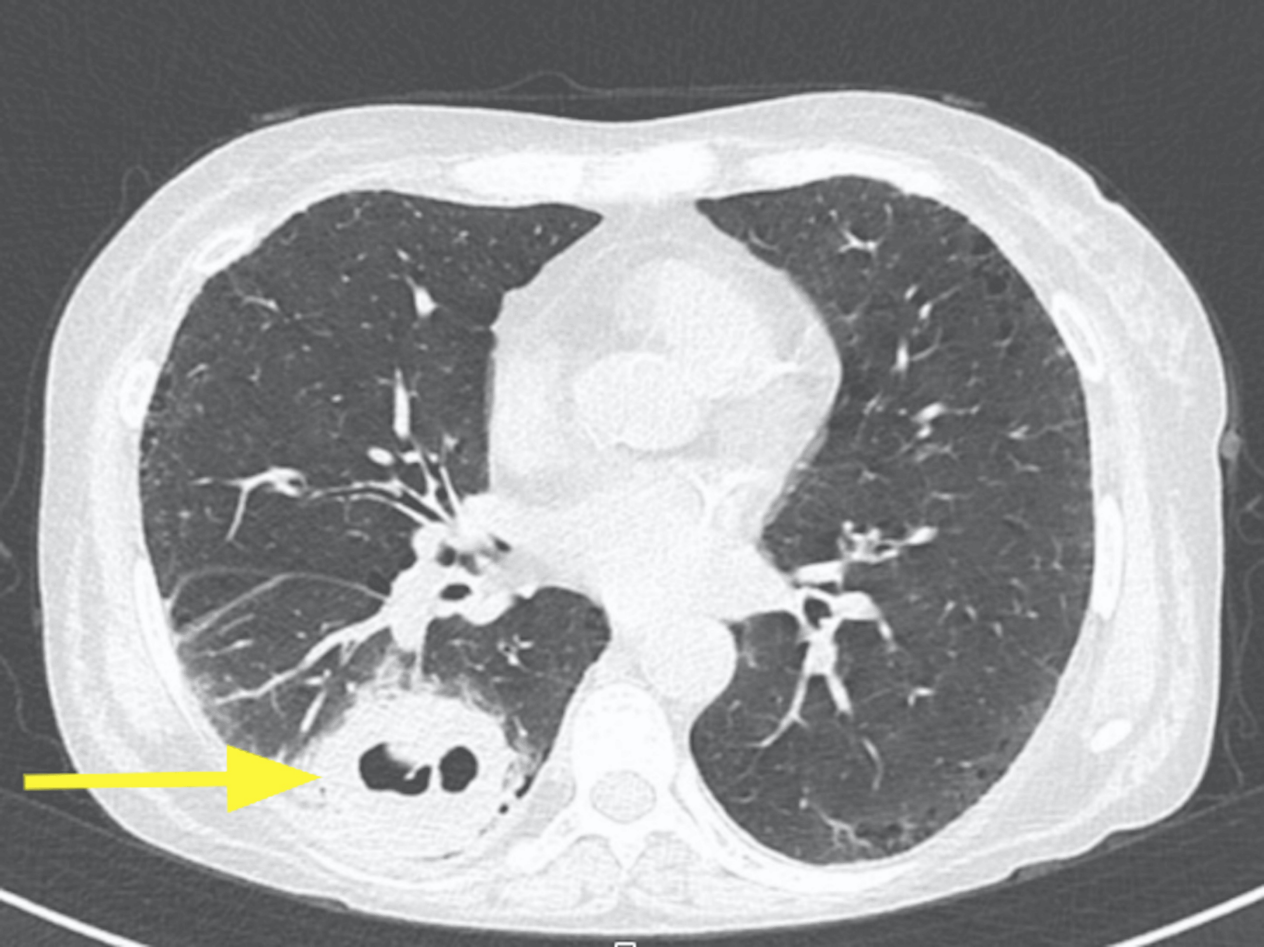
CT of the thorax revealing a mass in the basal segment of the lower right lobe (arrow) CT: computed tomography

**Figure 2:**
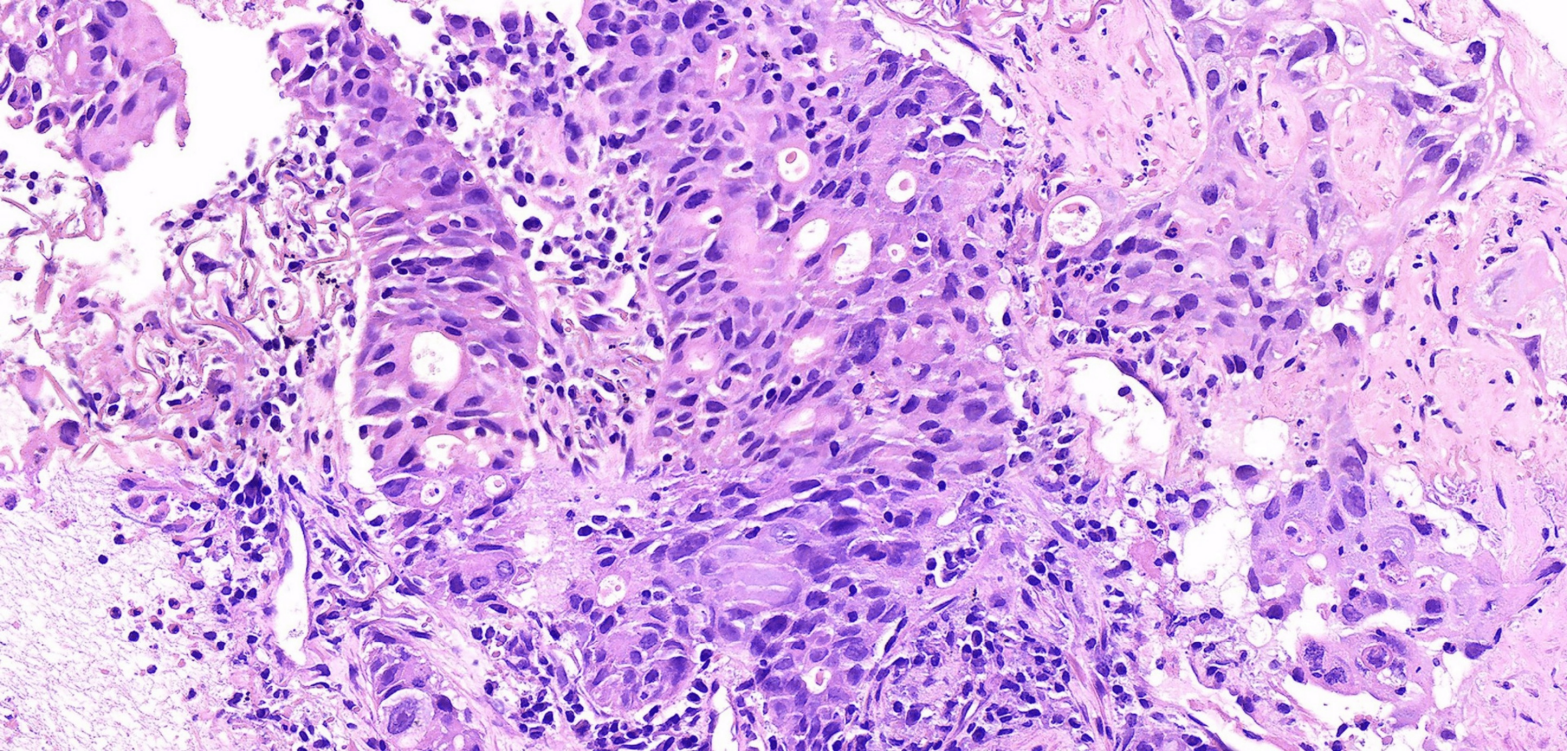
Poorly differentiated non-small cell lung carcinoma with squamous elements (slide stained with H&E, 40× magnification) H&E: hematoxylin and eosin

A computed tomography (CT) of the thorax was taken and revealed a 6.3 cm partially cavitated mass in the basal segment of the lower right lobe, abutting the pleura, accompanied by a proximal 2 cm nodule (Figure 1). A biopsy performed on February 1, 2022, on the right lung lesion, identified it as a poorly differentiated non-small cell lung carcinoma with squamous elements (Figure 2).

Treatment of the lung carcinoma commenced on February 24, 2022, with pembrolizumab. The patient also underwent a right lower lobectomy and lymphadenectomy on March 14, 2022. Treatment with pembrolizumab was halted due to the development of autoimmune pneumonitis between March and April 2023.

**Figure 3:**
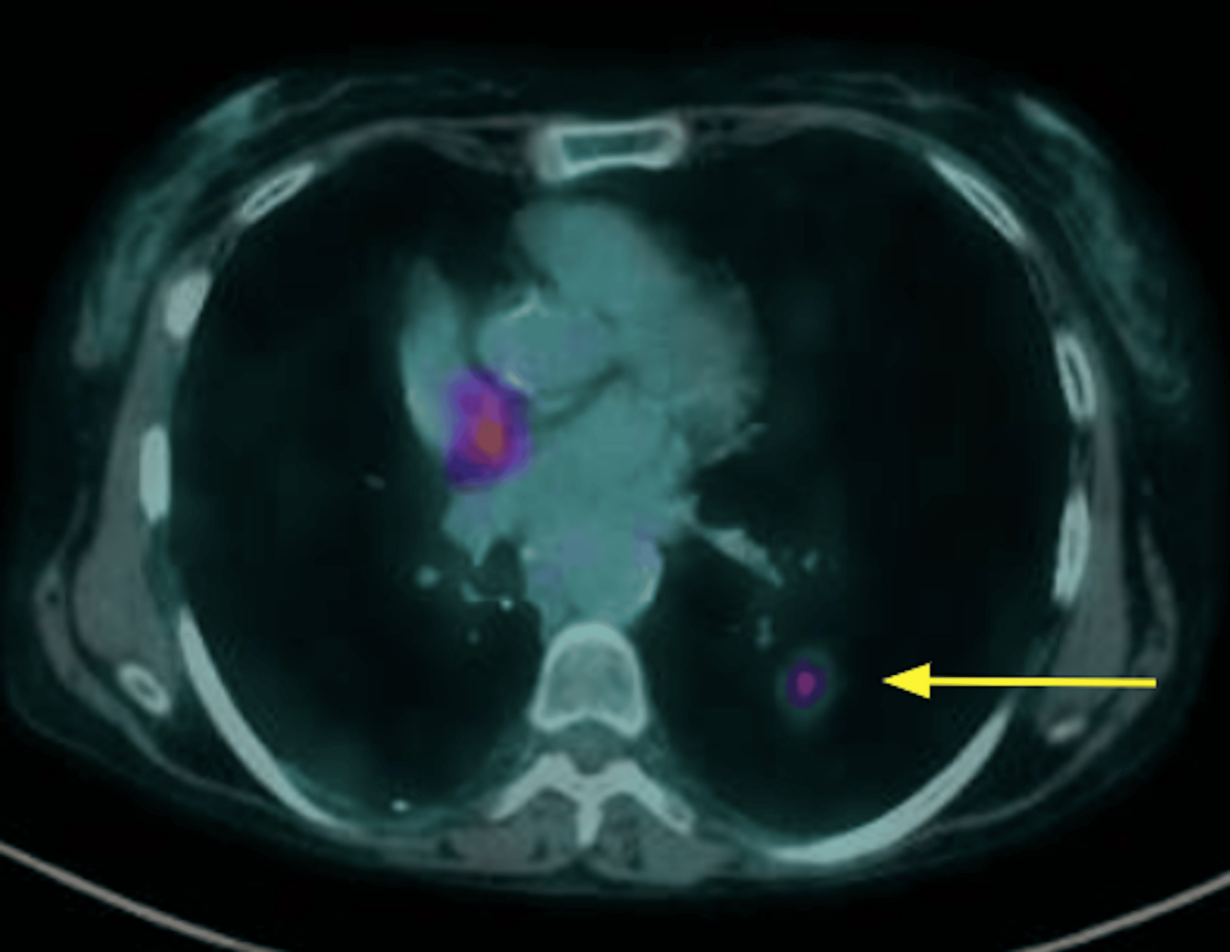
PET-CT showing progressive pulmonary disease (arrow) PET-CT: positron emission tomography, CT: computed tomography

A positron emission tomography (PET)-CT performed on April 25, 2023, revealed evidence of progressive pulmonary disease, prompting a biopsy of a nodule located in the posterior basal segment of the left lower lobe on May 2, 2023. The biopsy confirmed the diagnosis of adenocarcinoma (Figure 3). The patient subsequently underwent radiotherapy targeting the left lower lobe from May 19-29, 2023.

Molecular profiling via Foundation One testing on May 24, 2023, showed 0% programmed death-ligand 1 (PD-L1) expression, microsatellite stability, and the presence of mutations in BRAF (L597Q), EGFR (V786M), and PIK3CA (H1047R). On December 23, 2023, the patient was diagnosed with leptomeningeal recurrence, leading to the initiation of osimertinib therapy at a daily dose of 80 mg.

On January 17, 2024, the patient presented at the emergency department with generalized malaise, fatigue, and melena. She was receiving rivaroxaban 15 mg twice daily for a previously diagnosed deep vein thrombosis. Initial assessments showed hemodynamic stability, with abdominal examination revealing mild distension and minimal tenderness.

Laboratory investigations noted a decrease in hemoglobin from 8.4 to 6.5 g/dL over a week and thrombocytopenia with a platelet count of 34,000/mm³. These findings necessitated an endoscopic evaluation for gastrointestinal bleeding.

**Figure 4:**
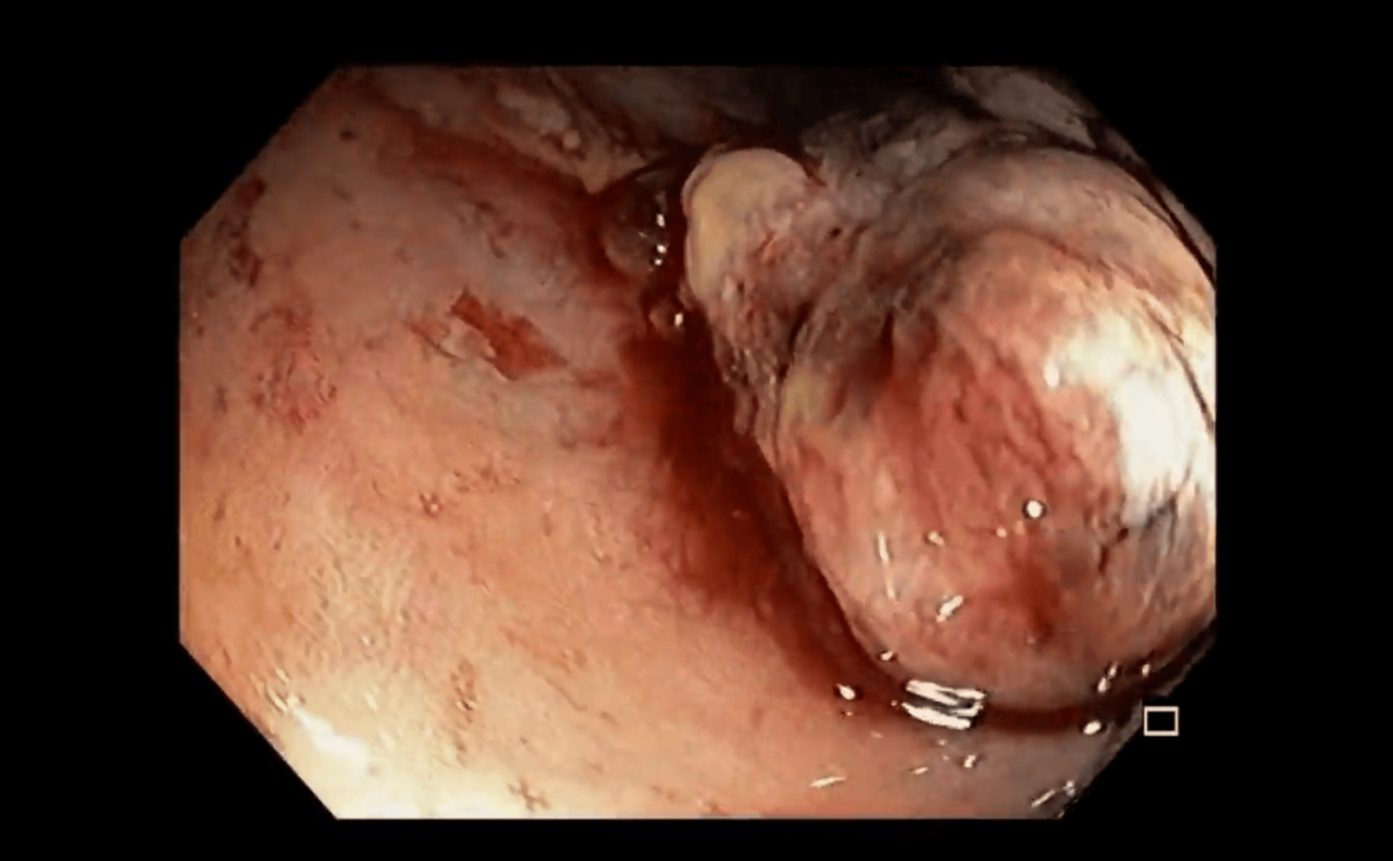
Colonoscopy showing bleeding metastasis

**Figure 5:**
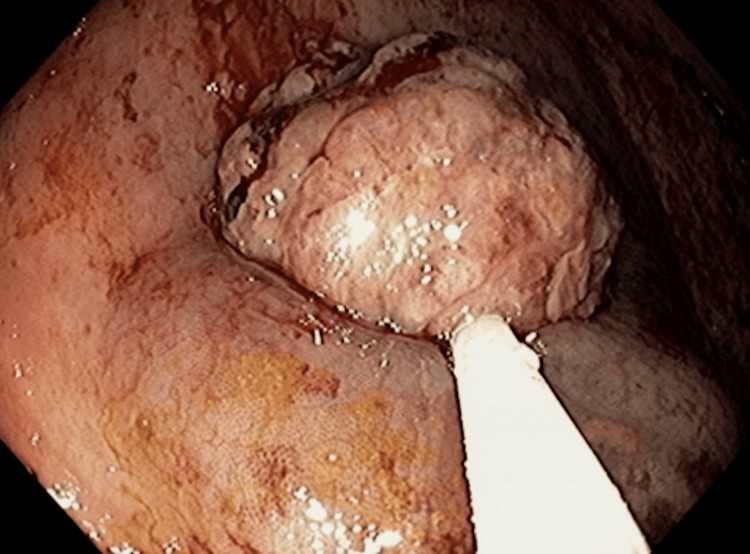
Mucosectomy for lesion in bloc resection

**Figure 6:**
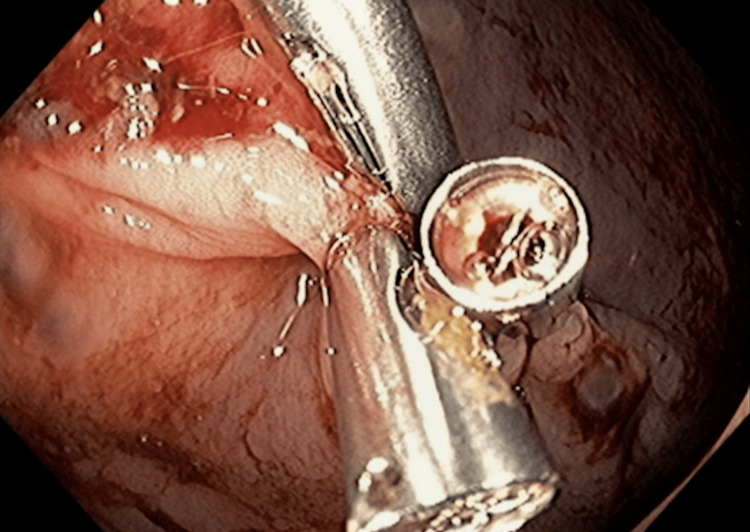
Three hemostatic clips applied after mucosectomy

**Figure 7:**
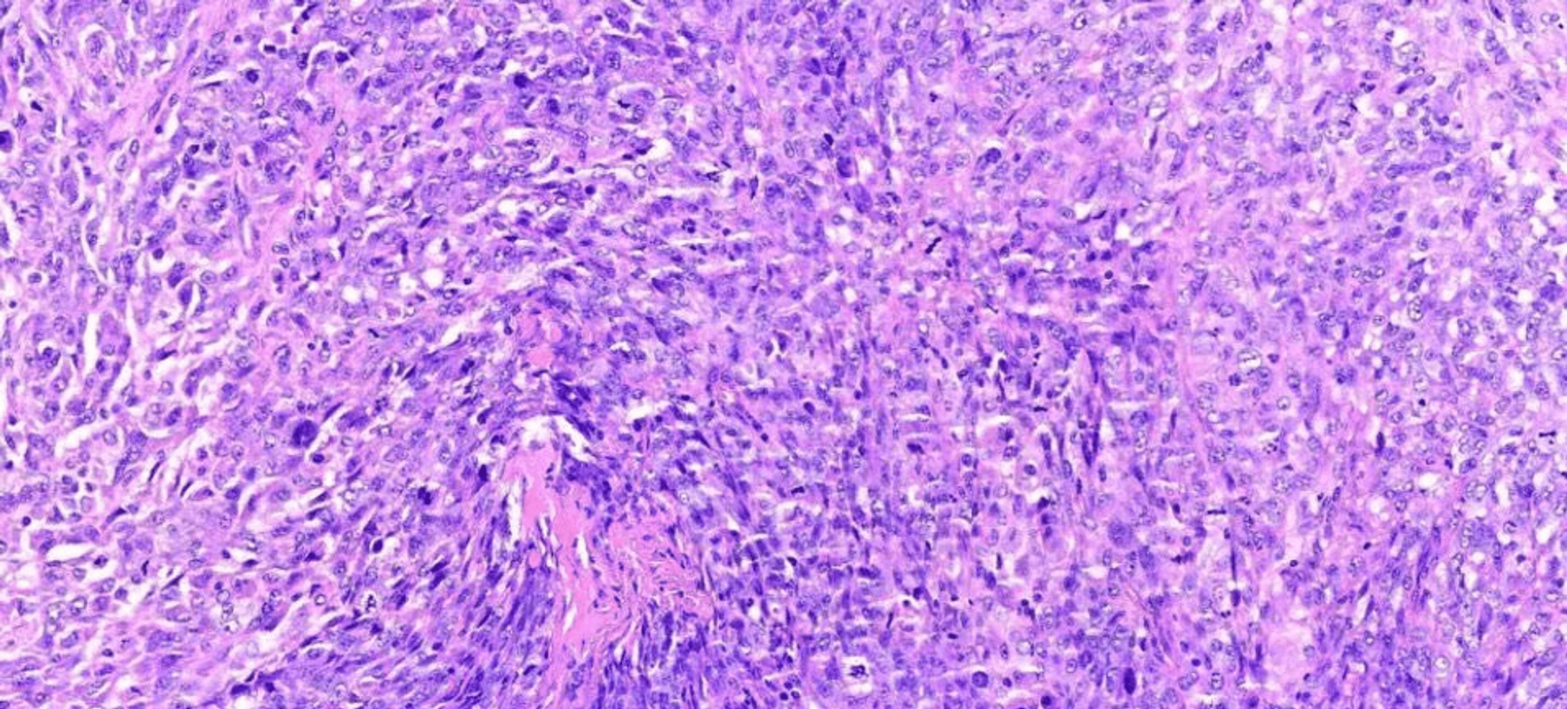
Poorly differentiated carcinoma with solid histoarchitecture, frequent mitoses, and occasionally abundant eosinophilic cytoplasm, with markedly pleomorphic, hyperchromatic nuclei and prominent nucleoli

**Figure 8:**
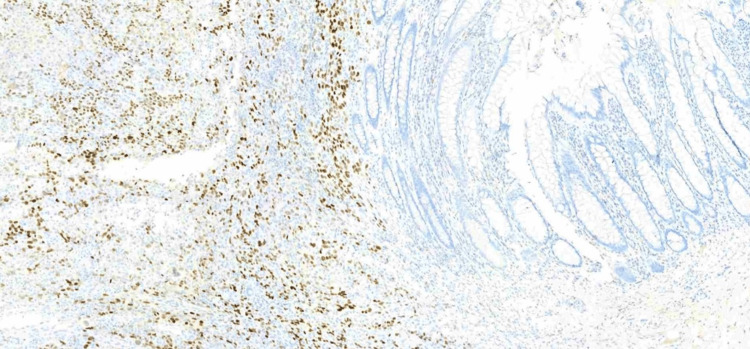
Immunohistochemistry: TTF-1 positive in the neoplasm and negative in the colonic mucosa

**Figure 9:**
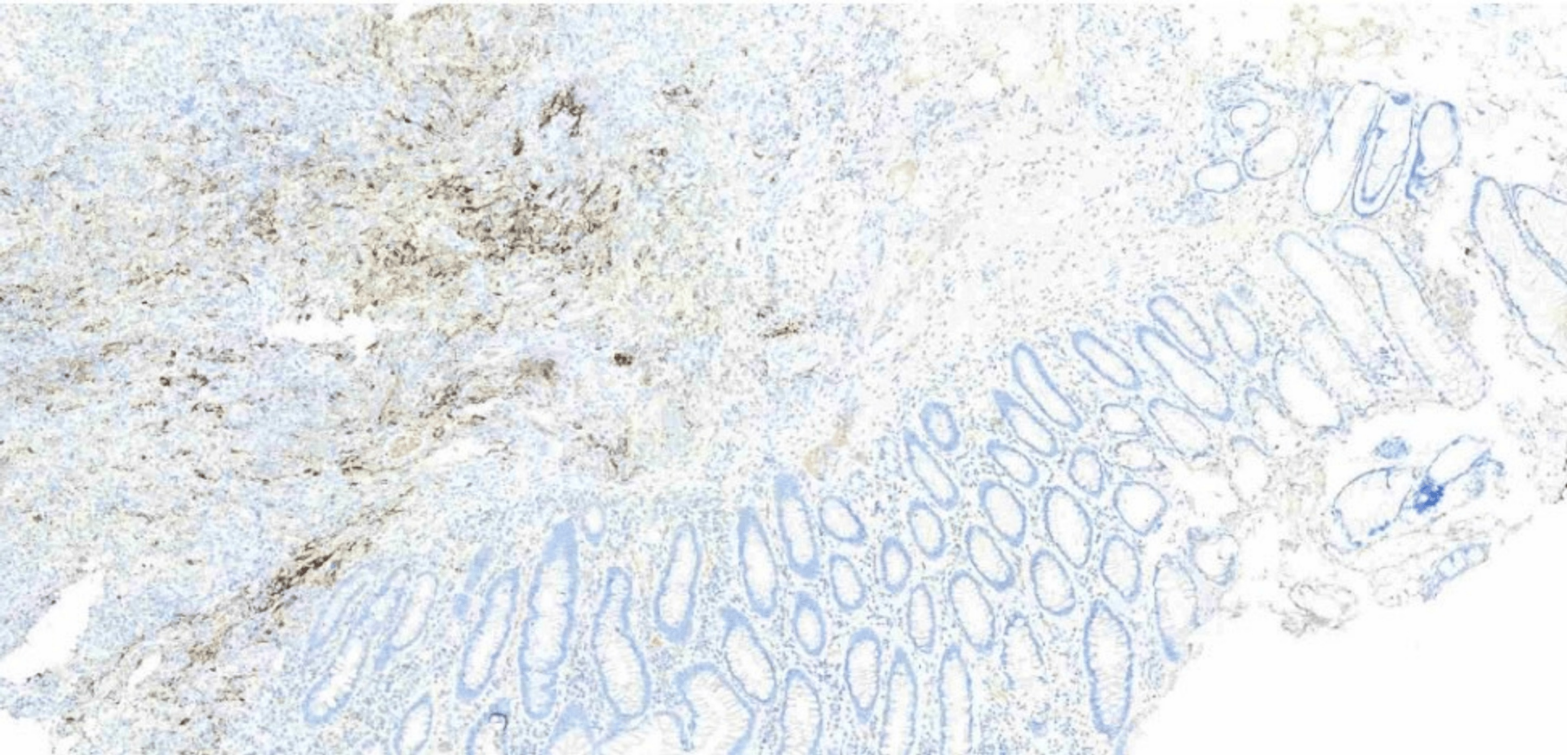
Immunohistochemistry: Napsin A positive in the neoplasm and negative in the colonic mucosa

**Figure 10:**
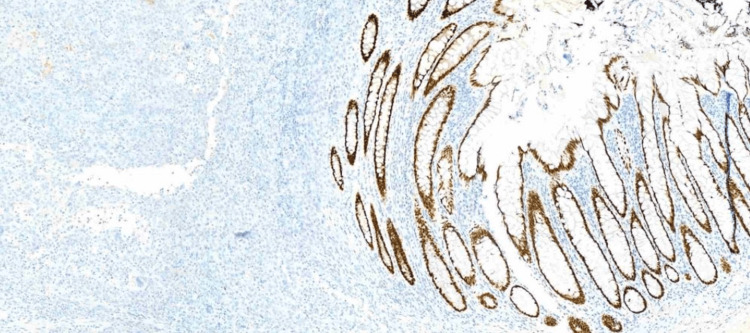
Immunohistochemistry: CDX2 negative in the neoplasm and positive in the colonic mucosa

On January 18, 2024, endoscopic examination disclosed a small hiatal hernia and mild erosive pangastritis. On January 19, 2024, a colonoscopy revealed a 3 cm sessile polypoid lesion in the ascending colon, exhibiting recent hemorrhage (Figure 4). Mucosectomy was performed for the lesion in bloc resection (Figure 5), and three hemostatic clips were applied (Figure 6). Pathological analysis of the resected tissue showed poorly differentiated malignant neoplasia with a predominantly solid arrangement infiltrating the submucosa, yet the muscularis propria remained uninvolved (Figure 7). Tumor cells were characterized by pleomorphism, hyperchromatic nuclei with conspicuous nucleoli, and frequent, atypical mitotic figures. Immunohistochemistry was positive for CK8/18, TTF-1, and Napsin A, and negative for p40, CDX-2, SATB2, and SOX-10 (Figures 8-10). These findings, coupled with the morphologic characteristics, established the diagnosis of metastatic lung adenocarcinoma.

The patient's post-procedural course was unremarkable, and she recovered smoothly following the endoscopic resection.

## Discussion

This case report illustrates a rare instance of metastatic lung adenocarcinoma presenting as a sessile polypoid lesion in the ascending colon. The rarity of colon metastasis from primary lung cancer [6,7] makes this case noteworthy and contributes significantly to the understanding of lung cancer metastasis.

Lung cancer commonly metastasizes to the brain, bones, liver, and adrenal glands; gastrointestinal metastases are uncommon [5]. The colon is an especially rare site for metastasis, with only a handful of cases reported. Documented rates of gastrointestinal metastasis from lung cancer are 0.4% to the stomach, 1.1% to the small intestine, and 0.5% to the colon [8]. This case underscores the need for clinicians to maintain a high index of suspicion for metastatic disease in patients with lung cancer presenting with gastrointestinal symptoms, even when such occurrences are rare.

Diagnosing metastatic lung cancer to the colon is challenging due to its rarity and non-specific clinical presentation. Most gastrointestinal metastases are diagnosed at an advanced stage due to asymptomatic progression, resulting in a poor prognosis [9]. In this case, the patient's history of chronic diseases and gastrointestinal symptoms initially obscured the underlying metastatic process. Identification of metastasis relied primarily on histopathological and immunohistochemical findings. The tumor's immunoprofile, positive for CK8/18, TTF-1, and Napsin A, and negative for gastrointestinal markers, was decisive in confirming the pulmonary origin of the adenocarcinoma.

Management of metastatic lung cancer to the colon involves multidisciplinary care, including surgical, oncological, and supportive measures. While endoscopic resection, as performed in this case, can be curative for isolated metastasis, widespread disease often necessitates systemic therapy [9]. Chemotherapy has been shown to impact survival outcomes in patients with metastatic lung cancer to the gastrointestinal tract [9,10]. The use of targeted agents, such as osimertinib in this patient with an EGFR mutation, represents the evolving landscape of personalized cancer therapy [11].

In this case, the resection of the colonic lesion primarily aimed to control active gastrointestinal bleeding rather than serve as a curative measure. At the time of the colorectal metastasis diagnosis, the patient was already polymetastatic with widespread disease, including leptomeningeal involvement. Therefore, the surgical intervention was palliative, addressing the immediate issue of bleeding and improving the patient's quality of life, while systemic therapy with osimertinib continued to manage the underlying metastatic disease.

The prognosis of lung cancer with gastrointestinal metastasis remains poor, with a median overall survival ranging from two to six months after the diagnosis of gastrointestinal involvement. Nonetheless, data remain limited due to the rarity of such cases. In a large autopsy review, Yoshimoto et al. reported an incidence of 0.5% and a median survival of 2.3 months after diagnosis [12]. Yang et al. described 10 patients with small bowel metastases and a median survival of 4.5 months, despite surgical or palliative treatment [13]. Earlier reports by Antler et al. [14] and McNeill et al. [15] also highlighted the aggressive course of these metastases, which frequently present with obstruction, perforation, or bleeding. Most patients have advanced disease and receive only palliative therapy. Surgical intervention, when feasible, may provide temporary symptom relief and, in rare cases, a modest survival benefit, particularly in patients with isolated intestinal metastasis and good performance status. Long-term follow-up data remain scarce, but recurrence and progression are common even after resection. This case adds valuable information to the limited literature, improving the understanding of prognosis and management strategies for similar presentations.

## Conclusions

This case highlights the importance of considering metastatic lung cancer in the differential diagnosis of colonic lesions, particularly in patients with a known history of lung cancer. It also emphasizes the role of comprehensive histopathological and immunohistochemical analyses in diagnosing atypical metastatic sites. As the therapeutic landscape for lung cancer continues to evolve, this case underscores the need for awareness of unusual metastatic patterns, facilitating timely diagnosis and appropriate management.
